# Seroprevalence of canine hepatitis in stray dogs in Nineveh Province, Iraq

**DOI:** 10.14202/vetworld.2020.2326-2329

**Published:** 2020-11-04

**Authors:** Zahraa Mustafa Al-Jumaa, Eva Aisser Ajaj, Mohammad Osamah Dahl

**Affiliations:** Department of Internal and Preventive Medicine, College of Veterinary Medicine, University of Mosul, Mosul 41002, Iraq

**Keywords:** alanine aminotransferase, alkaline phosphatase, aspartate aminotransferase, dogs, Hepatitis B virus

## Abstract

**Aim:**

The current study was conducted to explore evidence of hepatitis B virus (HBV) infection in dogs in Nineveh Province, Iraq.

**Materials and Methods:**

Serum samples of 78 dogs were used to (i) estimate levels of the antibodies against HBV through enzyme-linked immunosorbent assay and (ii) measure the activity of liver function enzymes.

**Results:**

Seropositive dogs for HBV constituted 9% of total tested dogs. The differences in seropositivity among males compared to females and among different ages were not statistically significant. Liver function enzymes analysis revealed a significant increase in the activity of alanine aminotransferase, aspartate aminotransferase, and alkaline phosphatase in seropositive dogs compared to those seronegative.

**Conclusion:**

Hepatitis B is evident in dogs in Nineveh Province, Iraq, with a significant impact on liver function in affected dogs. It is important to confirm this evidence through molecular tests.

## Introduction

Hepatitis B virus (HBV) is a hepatotropic DNA virus belongs to the family: Hepadnaviridae, genus: Orthohepadnavirus, and classified into four serotypes with 10 genotypes including different subtypes [1,2]. Although HBV infection requires species-specific host factors [3,4] and mainly affects human, the HBV and HBV-like virus can infect different types of mammals including chimpanzees, gibbons, gorillas, orangutans, woolly monkeys, pigs, and squirrels, with potential cross-species transmission among different hosts [5,6]. Recently, HBV-like virus has been identified in domestic cats [7]. An identical prototype has been detected in 10.8% sera collected from 390 household cats [8]. In these two studies, the virus was detected in cats affected with feline immunodeficiency virus, a virus close to human immunodeficiency virus that can infect human concurrently with HBV [7]. Moreover, the virus has been identified in 43% of cats affected with chronic hepatitis and 28% of those affected with hepatocellular carcinoma [9].

In attempts of studying HBV infection in human using animal models, HBV infection has not been supported after complementation of the primary hepatocytes of dogs with sodium taurocholate cotransporting polypeptide; a species-specific host factor [4]. However, in a study tested serum samples of different animal species for hepatitis B surface antigen, hepatitis B surface antibody, and antibody against hepatitis B core antigen using radioimmunoassays, dogs were among animals that showed positive reaction for hepatitis B surface antibody [10]. In a more recent study, 10% and 5.8% of tested domestic dogs showed positive reaction for antibody against hepatitis B core antigen and hepatitis B surface antigen, respectively, using enzyme-linked immunosorbent assay (ELISA) for serum samples [6]. In that study, the molecular tests indicated that 10% of domestic dogs were positive for the pre-S/S gene of HBV.

In Iraq, dogs are not usually adopted as pets for different traditional and religious reasons. On the other hand, stray dogs are common even inside urban areas. In this situation, if a cross-species transmission among different hosts is existed in HBV [5], and dogs are potentially infected with HBV or HBV-like virus [6], investigation of HBV in dogs is important. To the best of our knowledge, no study has investigated HBV infection in dogs in Nineveh Province, Iraq. On the other hand, the prevalence of HBV infection in humans in Mosul, the center city of Nineveh Province, was recently estimated at 76.31% using HBV surface antigen (HbsAg) as a marker for the infection [11].

The objective of the study was to explore evidence of HBV infection in dogs in Nineveh Province, Iraq.

## Materials and Methods

### Ethical approval

Ethical approval was not necessary for this study; however, serum samples have been collected according to the standard procedure for sample collection.

### Study area, dogs and period

A total of 78 different breeds of dogs from different areas from Nineveh Province, Iraq were used in this study. Dogs included male (n=45) and female (n=33) with different ages (mean=2.5 years old, min=1.5 months old, and max=6 years old). Serum samples were collected between October 2018 and October 2019.

### Laboratory examination

Serum samples collected from study dogs were used to estimate antibodies levels against HBV through ELISA and liver function enzyme activities including alanine aminotransferase (ALT), aspartate aminotransferase (AST), and alkaline phosphatase (ALP).

Canine HbsAg ELISA kit (SunLong Biotech Co., Ltd., China) was used to estimate antibodies levels against HBV. All quality control measures were taken into consideration. For instance, kit reagents were kept at room temperature, the automated microplate reader was standardized before the assay, and a fresh pipette tip was used for each sample to prevent cross-contamination. In addition, ELISA procedure was conducted according to the manufacturer’s instructions. In brief, (i) the test effectiveness was considered as the average value of the optical density (OD) for positive control ≥1.00, and for negative control ≤0.10; (ii) cutoff value was set at 0.2635 U/L, which was calculated according to the manufacture’s instruction as the following: Cutoff value=average value of negative control+0.15; (iii) serum was judged as canine HbsAg negative if the OD value was lower than the cutoff value, and positive if the OD value was equal or greater than the cutoff value. Finally, the OD was measured at a wavelength of 450 nm.

Liver function enzymes activities, that is, ALT (U/L), AST (U/L), and ALP (U/L), were estimated using Mindray BS-230 clinical chemistry analyzer (Shenzhen Mindray Bio-Medical Electronics Co., Ltd., China). Quality control measures were taken into consideration. That is, a fresh pipette tip was used for each sample and both reagents 1 and 2 of the kit, as well as estimation steps per the manufacture’s instruction.

### Statistical analysis

In this study, logistic regression was used to evaluate the odds of HBV infection according to the sex and age of the study dogs [12]. Two-sample Wilcoxon rank-sum (Mann–Whitney) test was used to evaluate the activity of ALT, AST, and ALP in seropositive dogs compared to those seronegative; as the variables for ALT, AST, and ALP were not normally distributed using Shapiro–Wilk W-test for normality and frequency distribution histogram [13]. Finally, in all analyses, p≤0.05 (two-tailed) was considered significant. Statistical analyses were performed using STATA 13.0 (StataCorp, College Station, TX, USA).

## Results

**Figure-1:**
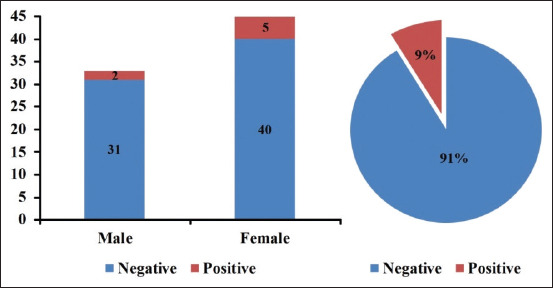
Seropositivity against canine hepatitis B virus antigen, and the frequency of the infection according to the sex of study dogs in Nineveh Province, Iraq.

A total of 7 dogs (9%) were indicated as seropositive for hepatitis B disease, including two males and five females (Figure-1). The intra-assay coefficient of variability was 7.04% for positive samples and 10.1% for negative samples, and the interassay coefficient of variability was 8.58%, indicating reliable results. The odds of the infection in females were 1.9 times higher than that of males; however, it did not reach the significant statistical level (i.e., 95% confidence interval=0.35, 10.67; p=0.45). The odds of the infection increased 1.5 times for each unit increase in the age; however, it did not reach the significant statistical level, too (i.e., 95% confidence interval=0.84, 2.61; p=0.17). Liver function enzymes analysis revealed a significant increase in the activity of ALT, AST, and ALP in seropositive dogs (130.07, 65.54, and 317.5 U/L, respectively; p<0.01), compared to those seronegative (Table-1) [14].

**Table-1:** Liver function enzymes activity in seropositive dogs for canine hepatitis B virus antigen, compared to those seronegative in Nineveh Province, Iraq.

Parameter	Reference value*	Mean±Standard error (95% confidence interval)		p-value

		Positive (n=7)	Negative (n=71)	
Alanine aminotransferase U/L	47±26	130.07±3.2 (122.15-137.99)	38.82±3.6 (31.60-46.03)	<0.01
Aspartate aminotransferase U/L	33±12	65.54±6.2 (50.47-80.62)	37.81±3.1 (31.47-44.15)	<0.01
Alkaline phosphatase U/L	66±36	317.5±92 (92.36-542.64)	61.36±5.3 (50.85-71.88)	<0.01

* Kaneko *et al.* [14]

## Discussion

The current study indicated that the hepatitis B infection is evident in dogs in Nineveh Province, Iraq. The seropositivity, however, was not different among males and females as well as among different ages. The evidence of HBV in dogs was not only explored through serological marker for HBV (i.e., HbsAg in this study) but also was liver function enzymes activity estimated as an indicator for liver damage. However, the number of study dogs was small, that is, 78 only, which is a limitation of this study. Thus, a study that confirms this result is required.

In this study, 9% of tested dogs were seropositive for canine HbsAg. This result is in line with the study of Vieira *et al*. [6] where 5.8% of dogs tested positive. The seropositivity in the current study could reflect HBV or HBV-like virus infections. One reason is that dogs could be exposed to the HBV infection from the environment potentially contaminated by infected humans [6,10]. Another reason is that the seropositive reaction is due to HBV-like virus that is adopted to infect dogs. However, molecular tests can confirm the gene.

Analysis of study data indicated that the differences in seropositivity among males compared to females and among different ages were not statistically significant. A potential explanation is that the association between HBV infection and sex as well as age of dogs is real; however, the sample size of the current study was too small to declare that the observed association is statistically significant. However, results of the current study are in line with the study of Lanave *et al*. [8] where the hepadnavirus infection in cats is not associated with sex and age. Further studies with probability sampling are required to confirm such results.

In this study, seropositive dogs showed a significant increase in the activity of liver function enzymes including ALT, AST, and ALP compared to seronegative dogs. Elevation of these enzymes indicates presence of liver disease [15]. That is, infected hepatocytes are targeted by cellular immunity, particularly cytotoxic T lymphocytes, to eliminate the infection [16], and eventually damage the liver. The results of the current study are in line with Abulude *et al*. [17] study who found that seropositive patients to HBV showed elevation of ALT, AST, and ALP activities levels.

## Conclusion

This study indicated that hepatitis B infection is evident in dogs in Nineveh Province, Iraq, with a significant impact on liver function in affected dogs. It is important to confirm this evidence through molecular tests.

## Authors’ Contributions

All authors contributed in the conceptualization. ZMA and EAA collected the samples and conducted the laboratory examinations. MOD performed data organization, software and formal data analysis, as well as writing: Original draft and editing. All authors reviewed and approved the final manuscript.
